# Pharmacogenomics: methods and protocols

**DOI:** 10.1038/sj.bjc.6603137

**Published:** 2006-05-16

**Authors:** C Lai

**Affiliations:** 1 University College London, London, UK

Pharmacogenetics is about how the genetic inheritance of a person affects his response to medicine, examining relationships between genes and drug metabolism, drug response and adverse effects. Despite a reasonably long history of this field, which took off in the 1950s, success stories in cancer pharmacogenetics were rather lacking, with the most quoted examples being thiopurine methyltransferase (TPMT) genotype affecting the metabolism of mercaptopurine, and association between the promoter polymorphism in UDP-glucuronosyltransferase 1A1 gene and irinotecan induced adverse drug reactions.

In the last decade, the generation of a huge amount of genetic information by the Human Genome Project and the HapMap project, accompanied by the rapid improvement in genotyping technologies, have fuelled an increase in genetics studies on the aetiology of common traits. These same reasons also enable pharmacogenetic studies to move from mostly single gene based to genome-wide investigations. Reflecting this gain in scale, the term ‘pharmacogenomics’ is now often used. Together with the development of targeted cancer therapy with drugs like Glivec (Imatinib), Iressa (Gefitinib) and Herceptin (Trastuzumab), pharmacogenomics is set to play a bigger role in both understanding cancer drug mechanisms and in clinical practice.

*Pharmacogenomics*: *methods and protocols*, is one of the latest additions to the long running ‘Methods on Molecular Biology’ series. This book aims to introduce the latest technology in complex genetics to its readers, presumably bench-based scientists who are interested in recreating the experimental system for pharmacogenomic studies in their laboratory. The opening chapter provides a historical overview of the field and is followed by 12 chapters of protocols. The last section deals with the management of pharmacogenomic information.

The core of this book is the protocols, which are divided into two groups. The first group contains four chapters on functional analysis of gene variations, with three of them describing methodologies in investigating allele specific expression. The second part covers a range of genotyping techniques, from fluorescence-based microsatellite typing to SNP typing methods including pyrosequencing, Taqman and MALDI-TOF (Sequenom). Each of these chapters begins with an abstract and a brief introduction explaining the theoretical basis of the system, before launching into the detailed description of materials and methods. The protocols are concise and well written – in most cases enough instructions are provided for the reader to follow the experiment without the help of supplementary information. Their value, however, depends largely on the technique being described. The protocols are most useful for complicated assays like the Haplotype-specific Chromatin Immunoprecipitation, where multiple molecular biology techniques has to be carried out and the fine-tuning of experimental parameters vital to eventual success. Genotyping techniques, on the other hand, are often highly standardised, and instruction manuals can be obtained from the company manufacturing the genotyping equipment, making some of the protocols less interesting.

What distinguishes this book from the standard protocols, however, is the Notes section of each chapter, where the ‘tricks’ of the experimental system are discussed. The authors of this book are leading experts in complex genetics and pharmacogenetics, and their experience in applying particular techniques is the most valuable information for the readers. The tips they provided are very specific and often pinpoint both practical details (down to the particular type of flask to use) and issues in good experimental design (e.g. suggesting appropriate controls). By following them the readers can get a better grasp of the critical steps in the each procedure and hence have a higher chance of success when setting them up.

Most of the protocols contain a short description of the expected output from the experiment, often consists of a figure or a screenshot. It would be beneficial to expand this section and include more information on troubleshooting.

I suspect the majority of the readers of *Pharmacogenomics: methods and protocols* will only need a few of the protocols described. In the ideal world a book like this would be helpful when deciding on the particular technology at the beginning of a pharmacogenomic study. Practically though, the choice often depends on the availability of machinery and expertise in the laboratory rather than any other factor. So all in all, this book is a fine collection of protocols describing state of the art methods in functional and complex genetics that can be used in pharmacogenomics. While it might not become a most treasured item for individual scientists, it is certainly a good reference book to have in one's institute library.

